# Role of metabotropic glutamate receptor 5 signaling and homer in oxygen glucose deprivation-mediated astrocyte apoptosis

**DOI:** 10.1186/1756-6606-6-9

**Published:** 2013-02-14

**Authors:** Maryse Paquet, Fabiola M Ribeiro, Jennifer Guadagno, Jessica L Esseltine, Stephen SG Ferguson, Sean P Cregan

**Affiliations:** 1 J. Allyn Taylor Centre for Cell Biology, Robarts Research Institute, Department of Physiology and Pharmacology, The University of Western Ontario, 100 Perth Drive, London, ON, N6A 5K8, Canada; 2 Departamento de Bioquimica e Imunologia, ICB, Universidade Federal de Minas Gerais, Belo Horizonte, Brazil

**Keywords:** Astrocyte, Metabotropic glutamate receptor, Cell death, Inositol phosphate, Oxygen glucose deprivation, Homer

## Abstract

**Background:**

Group I metabotropic glutamate receptors (mGluR) are coupled via Gα_q/11_ to the activation of phospholipase Cβ, which hydrolyzes membrane phospholipids to form inositol 1,4,5 trisphosphate and diacylglycerol. In addition to functioning as neurotransmitter receptors to modulate synaptic activity, pathological mGluR5 signaling has been implicated in a number of disease processes including Fragile X, amyotrophic lateral sclerosis, multiple sclerosis, Alzheimer’s disease, Parkinson’s disease, Huntington’s disease, epilepsy, and drug addiction. The expression of mGluR5 in astrocytes has been shown to be increased in several acute and chronic neurodegenerative conditions, but little is known about the functional relevance of mGluR5 up-regulation in astrocytes following injury.

**Results:**

In the current study, we investigated primary mouse cortical astrocyte cell death in response to oxygen glucose deprivation (OGD) and found that OGD induced both necrotic and apoptotic cell death of astrocytes. OGD resulted in an increase in astrocytic mGluR5 protein expression, inositol phosphate formation and extracellular regulated kinase (ERK1/2) phosphorylation, but only inositol phosphate formation was blocked with the mGluR5 selective antagonist MPEP. Cortical astrocytes derived from mGluR5 knockout mice exhibited resistance to OGD-stimulated apoptosis, but a lack of mGluR5 expression did not confer protection against necrotic cell death. The antagonism of the inositol 1,4,5 trisphosphate receptor also reduced apoptotic cell death in wild-type astrocytes, but did not provide any additional protection to astrocytes derived from mGluR5 null mice. Moreover, the disruption of Homer protein interactions with mGluR5 also reduced astrocyte apoptosis.

**Conclusion:**

Taken together these observations indicated that mGluR5 up-regulation contributed selectively to the apoptosis of astrocytes via the activation of phospholipase C and the release of calcium from intracellular stores as well as via the association with Homer proteins.

## Background

Several neurotransmitter receptors are expressed in astrocytes including the G protein-coupled receptors (GPCRs) for the excitatory neurotransmitter glutamate. The metabotropic glutamate receptors (mGluRs) comprise the family C of GPCRs, which are characterized by sequence similarities in their seven transmembrane spanning helical domains and large extracellular amino-terminal venus fly trap domain [1-3]. Eight distinct mGluRs have been identified and are divided into three subgroups based on sequence homology and G protein coupling-specificity. Group I mGluRs (mGluR1 and mGluR5) are predominantly coupled to the activation of phospholipase C (PLC) via Gα_q/11_, whereas Group II (mGluR2 and mGluR3) and Group III (mGluR4, mGluR6, mGluR7 and mGluR8) mGluRs negatively regulate adenylyl cyclase via Gα_i_. Group I mGluR stimulation leads to Gα_q/11_-mediated intracellular activation of second messenger pathways, βγ activation of ion channels, as well as stimulation of G protein-independent pathways [2-4]. Pharmacological and biochemical experiments have shown that mGluR5 is the predominant group I mGluR subtype expressed in cultures of brain astrocytes [5,6] although mGluR1 is also expressed in astrocytes in the spinal cord [7].

Astrocytic mGluR5 has been shown to play key roles in glia-neuron interactions, regulation of glutamate reuptake, and the coupling of the neurovasculature to neuronal activity [8-10]. However, the roles of mGluR5 in both astrocytic and neuronal survival following injury are poorly understood. In neurons, Group I mGluRs have been reported to both protect against and exacerbate cell death, depending on the model of toxicity employed, the neuronal subtype involved, and prior activation state of the receptors themselves [11-17]. The variety of signaling pathways activated by mGluR1/5 may explain these observations. Group I mGluRs coupled to Gα_q/11_ proteins stimulate the activation of PLC resulting in diacylglycerol and inositol-1,4,5-triphosphate (InsP3) formation. By interacting with InsP3 receptors, InsP3 induces calcium release from intracellular stores potentially leading to inappropriate cell death [18]. On the other hand, mGluR1/5 stimulation also leads to activation of other signaling pathways important for cell survival/proliferation, such as extracellular signal-regulated kinases 1/2 (ERK1/2) and AKT [19,20]. Consistent with these opposing effects on cell survival, the mGluR1/5 scaffold protein Homer has been shown to promote the activation of both pro- and anti-survival signaling pathways [19,21].

Group I mGluR expression is up-regulated in models of epilepsy, brain injury, amyotrophic lateral sclerosis, and multiple sclerosis [22-25]. Alterations in glutamate signaling are also known to play a prominent role in ischemic brain injury and both apoptotic and necrotic astrocytes have been observed following *in vivo* ischemic insults [26-30]. However, very little is known about the functional consequences of elevated mGluR5 expression on astrocyte survival following injury. Therefore, in the present study, we utilized an *in vitro* model of ischemia/reperfusion, oxygen glucose deprivation (OGD), to evaluate the potential role of alterations in mGluR5 protein expression following the injury of astrocytes. We find that OGD induces astrocytic cell death by both apoptotic and necrotic mechanisms and results in a rapid increase in mGluR5a expression that is independent of gene transcription. The observed OGD-induced apoptotic cell death is associated with increased mGluR5-dependent inositol phosphate formation and is reduced in astrocytes isolated from mGluR5 knockout mice. Moreover, OGD-induced apoptotic death of cortical astrocytes was reduced following IP3 receptor antagonism and Tat-Homer peptide-mediated interference of Homer interactions with mGluR5. Taken together, these observations indicate that upregulation of mGluR5 expression contributes to OGD-induced cell death via a Gα_q/11_ and Homer mediated mechanism.

## Results

### OGD induces both necrotic and apoptotic cell death of cortical asrtocytes

**Figure 1:**
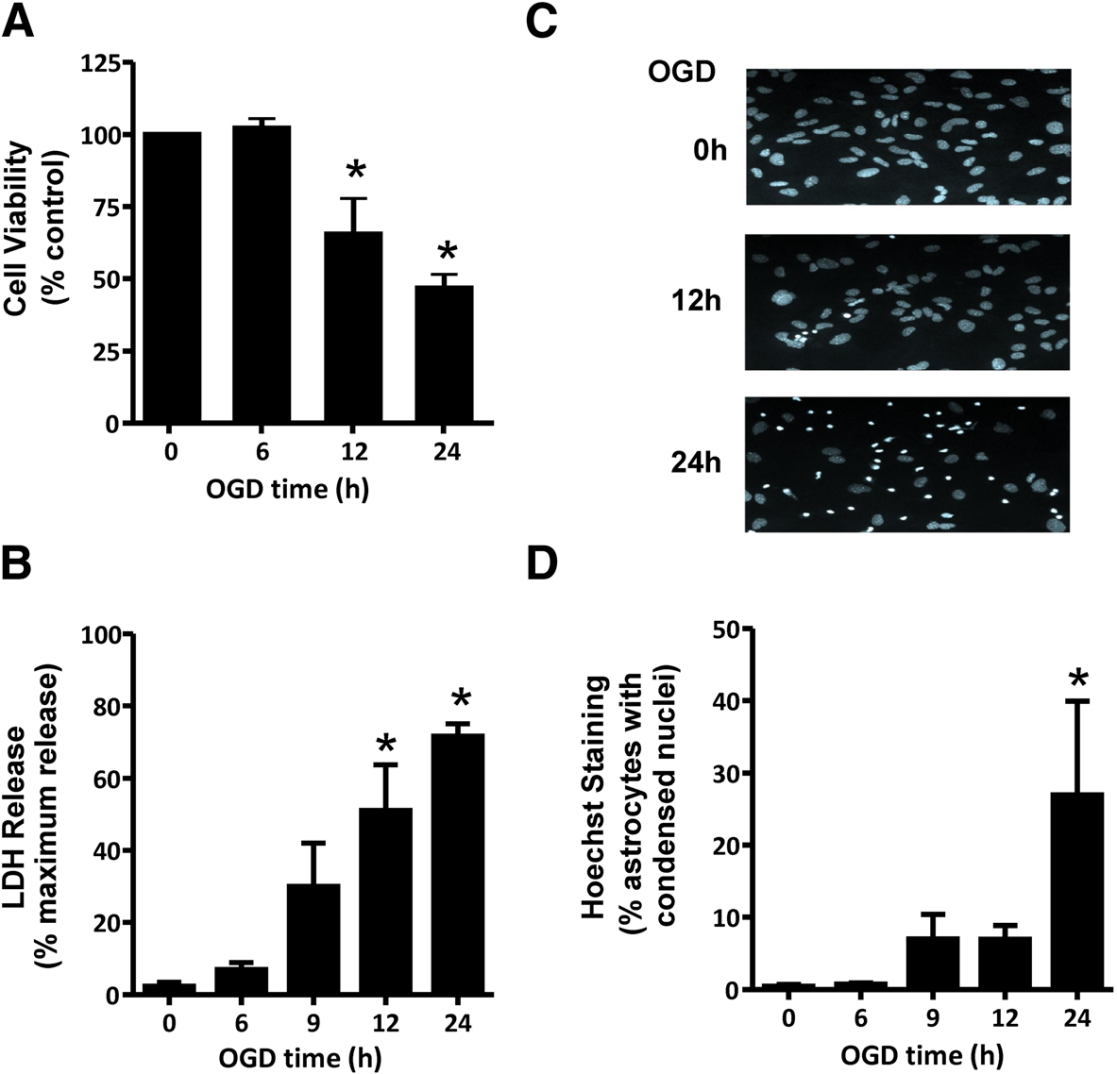
**OGD induces both necrotic and apoptotic cell death in cultured cortical astrocytes. A**) Cell viability of cultured cortical astrocytes, as assessed by the metabolic MTT assay following 0-24 h OGD followed by 24 h reperfusion. **B**) Necrotic cell death measured by lactate dehydrogenase (LDH) assay following 0-24 h OGD followed by 24 h reperfusion. **C**) Representative photomicrographs of primary CD1 cortical astrocytes cultures that were OGD treated for 0, 12, and 24 hours followed by 24 h reperfusion and stained with Hoescht dye to reveal cells dying by apoptosis as evidenced by condensed chromatin/ pyknotic nuclei. **D**) Quantification of apoptotic CD1 astrocytic cell death following 0-24 h OGD followed by 24 h reperfusion. All data represents the mean ± SD of 5 independent experiments and were analyzed by ANOVA followed by Dunnet’s post-hoc testing, * p<0.05 versus untreated control cultures.

To assess the effects of OGD on cortical astrocyte cell death, primary CD1 mouse cortical astrocytic cultures were subjected to an *in vitro* model of OGD to mimic ischemia (no oxygen and no glucose) for periods varying from 0 to 24 h followed by a 24 h reperfusion period (normal oxygen and glucose conditions). Under these conditions, astrocytes survived OGD treatment for 6 h (Figure 1A). However, further incubation without glucose and oxygen (12-24 h) led to a decrease in metabolic activity (Figure 1A). Both necrotic cell death as measured by the release of LDH by the dying astrocytes (Figure 1B) and apoptotic cell death as indicated by chromatin condensation observed in Hoechst-stained nuclei (Figure 1C) was induced following a 24 h OGD exposure time (Figure 1D). Thus, the reduction in astrocytes viability in response to OGD was associated with a mix of necrotic and apoptotic astrocytic cell death.

### OGD-induced increase in mGluR5 protein expression correlates with an increase in mGluR5-dependent inositol phosphate signaling

**Figure 2:**
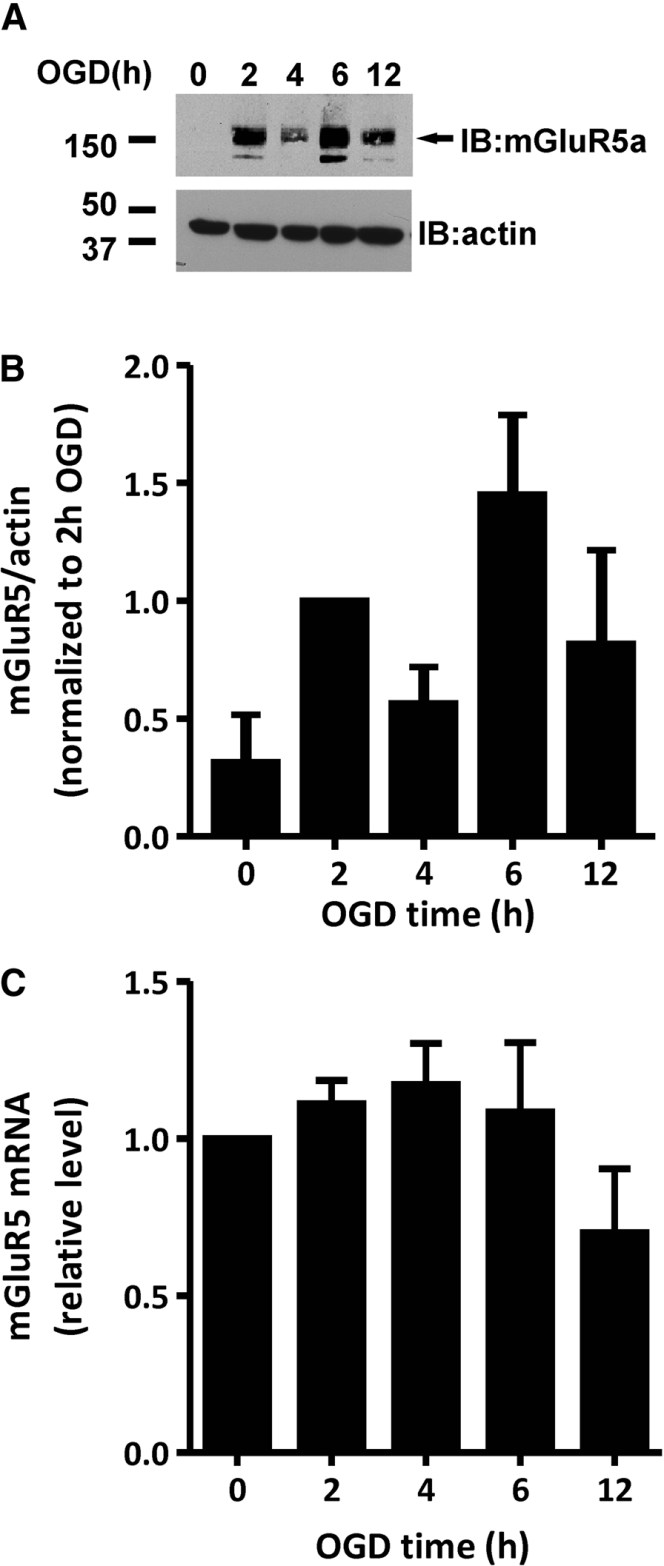
**mGluR5a protein expression is increased in cortical astrocytic cultures in response to OGD. A**) Representative immunoblot showing increases in mGluR5a protein expression at 0, 2, 4, 6, and 12 h following OGD treatment. **B**) Quantification of mGluR5a protein expression in primary CD1 mouse cortical astrocytes following 0-12 h OGD. Data is normalized to mGluR5a protein expression at 2 h. Data represents the mean ± SD of 4 independent experiments. **C**) Real time RT-PCR analysis shows no significant change in mGluR5 mRNA levels in cortical astrocytes after OGD. mGluR5 mRNA levels were normalized to mitochondrial S12 transcript levels and data represents mean ± SD from three independent experiments.

Cortical mouse astrocytes primarily express mGluR5a [5,6] and Group I mGluR expression have been previously shown to be up-regulated in models of brain injury, epilepsy, amyotrophic lateral sclerosis, and multiple sclerosis [22-25]. Therefore, we examined whether mGluR5a protein expression was altered following OGD treatment of primary cortical mouse astrocytes. As determined by immunoblotting for mGluR5a, the levels of mGluR5a expression in astrocytic cultures was not detectable by immunoblot in primary CD1 mouse cortical astrocytes, but OGD treatment (2-12 h) resulted in increased mGluR5a expression (Figure 2A and B). However, the increase in mGluR5a expression was not correlated with an increase in mGluR5a mRNA expression as determined by RT-PCR (Figure 2C) suggested that either mGluR5 turnover or mGluR5 protein translation was altered as a consequence of OGD.

### OGD-induced increase in mGluR5 protein expression correlates with an increase in mGluR5-dependent inositol phosphate signaling

**Figure 3:**
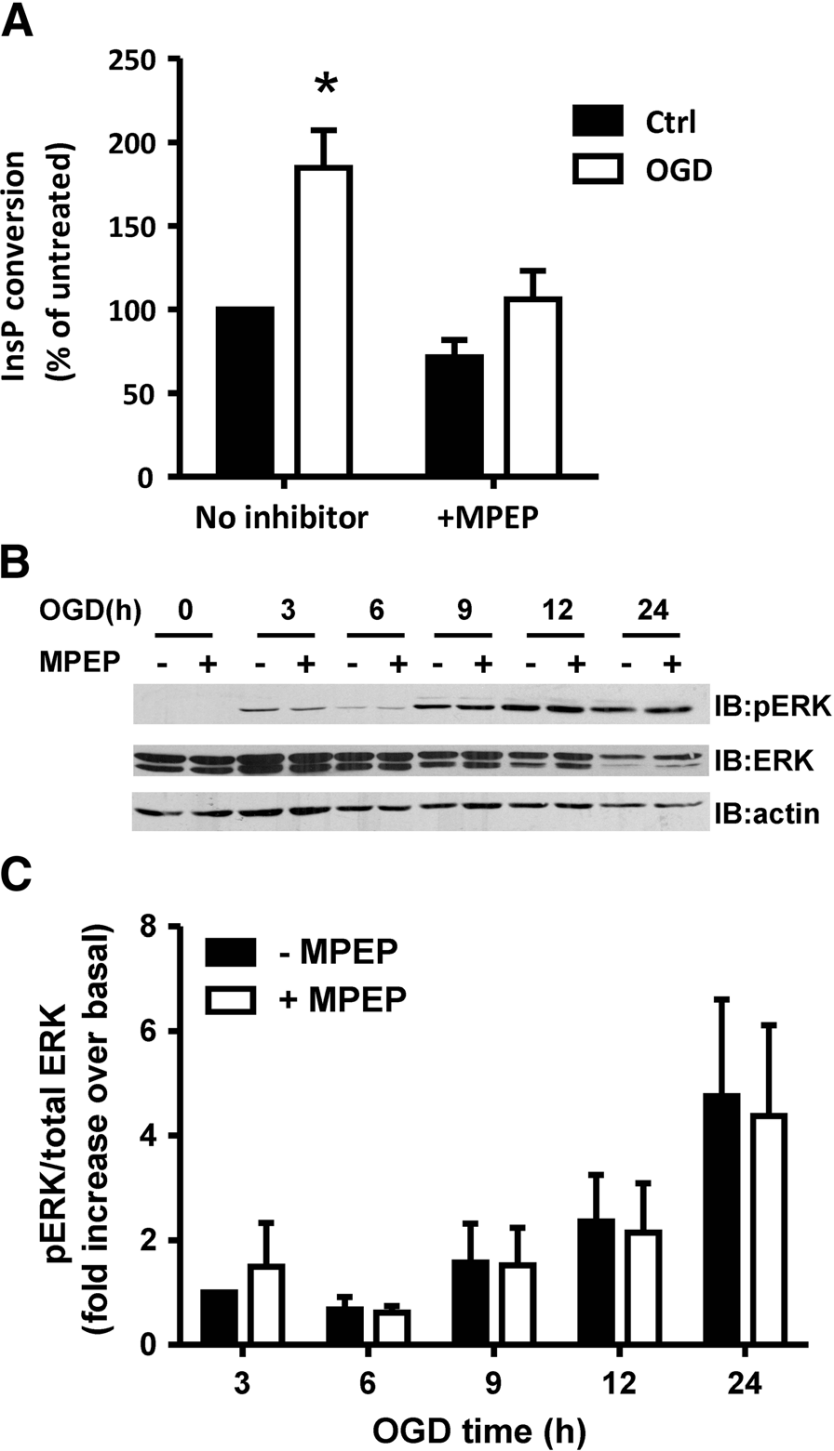
**Effect of OGD on InsP formation and ERK1/2 phosphorylation in cortical astrocytes. A**) Inositol phosphate (InsP) formation in primary CD1 mouse cortical astrocytes prior to (Ctrl) and following OGD for 4 h in the presence and absence of the mGluR5 inverse agonist MPEP (100 μM). Bonferroni post-hoc tests were performed on three independent experiments (* p<0.05). **B**) Representative phosph-ERK1/2, total ERK1/2 and actin blots of primary CD1 mouse cortical astrocytes following 0-24 h OGD followed by 24 h reperfusion treated in the absence and presence of the mGluR5 inverse agonist MPEP (100 μM). **C**) Quantification of ERK1/2 phosphorylation in primary CD1 mouse cortical astrocytes following 0-24 h OGD followed by 24 h re-oxygenation treated in the absence and presence of the mGluR5 inverse agonist MPEP (100 μM). Data was normalized to total ERK1/2 protein expression. Data represents the mean ± SD of 4 independent experiments.

Because mGluR5a protein expression was increased by OGD, we assessed whether mGluR5-dependent inositol phosphate (InsP) formation and ERK1/2 phosphorylation was altered in response to OGD. As shown in Figure 3A, InsP formation in cortical astrocytes following 4 h OGD was significantly increased compared to non-ischemic cultures (0 h OGD). The observed increase in InsP formation in response to OGD was blocked by treating the astrocytic cultures with the mGluR5-specific inverse agonist MPEP (100 μM) (Figure 3A). OGD treatment of cortical astrocyte cultures for increasing periods of time (3-24 h) resulted in a treatment and time-dependent increase in ERK1/2 phosphorylation (Figure 3B). However, unlike what was observed for InsP formation following OGD, MPEP treatment (100 μM) of the astrocytic cultures did not inhibit OGD-induced increases in ERK1/2 phosphorylation (Figure 3C). These data suggested that the mGluR5 receptors induced by OGD contributes to increased InsP formation, but that increases in ERK1/2 activity induced by OGD likely occurred independently of mGluR5.

### mGluR5 promotes apoptotic, but not necrotic astrocytic cell death

**Figure 4:**
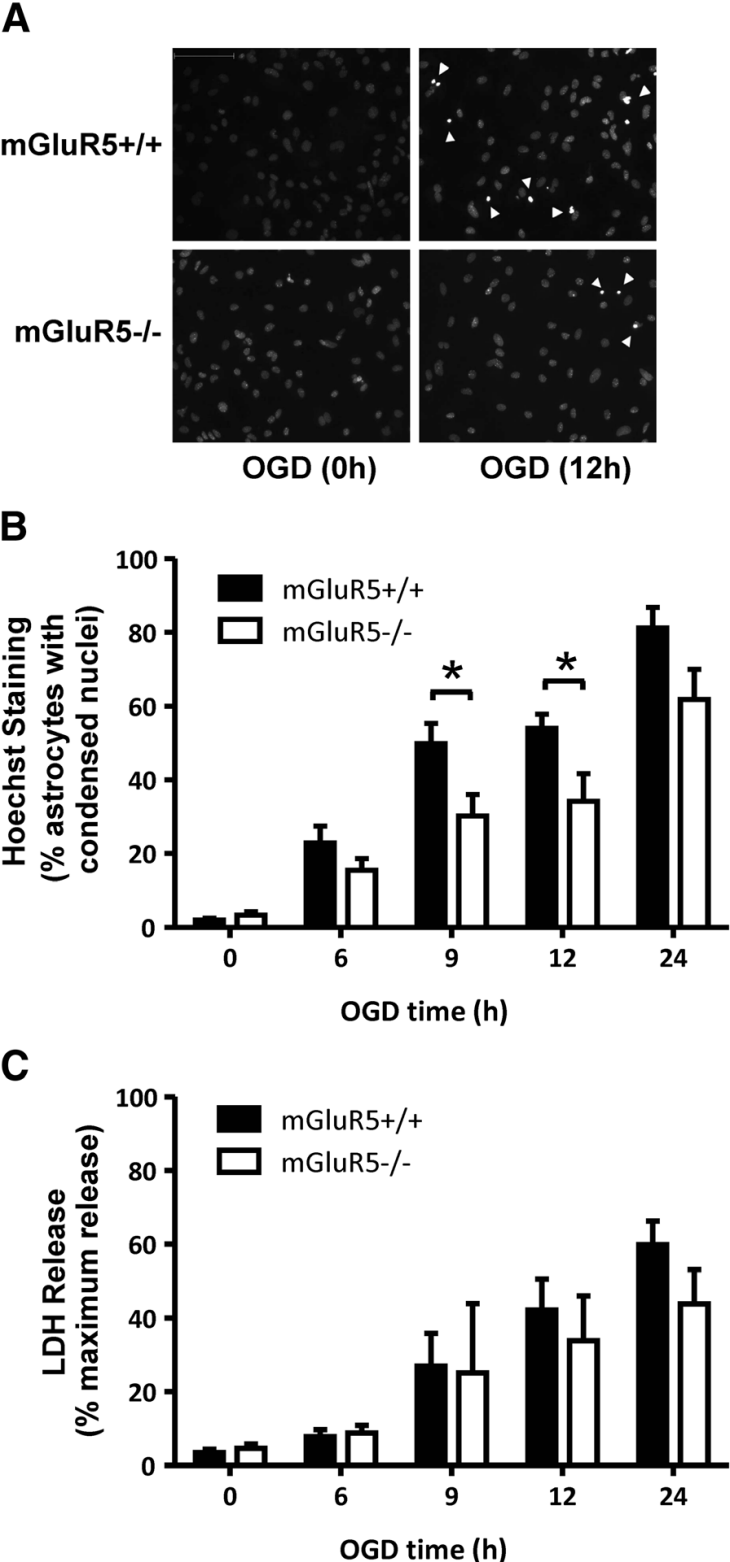
**OGD-mediated apoptotic and necrotic cell death in wild type and mGluR5 null cortical astrocytes. A**) Photomicrographs of primary cortical astrocytes derived from wild-type C57BL/6 (mGluR+/+) and mGluR5 knockout (mGluR5-/-) littermate embryos that were OGD treated for 0, 12, and 24 hours followed by 24 h re-oxygenation and stained with Hoescht dye to reveal cells dying by apoptosis as evidenced by condensed chromatin/pyknotic nuclei. **B**) Quantification of apoptotic death of astrocytes derived from mGluR5-/- and mGluR5+/+ embryos in response to 0-24 h OGD followed by 24 h re-oxygenation. Data represent the mean ± SD of 5-8 independent experiments. **C**) Necrotic cell death measured by LDH assay following 0-24 h OGD followed by 24 h re-oxygenation in cortical astrocytes derived from mGluR5-/- and mGluR5+/+ embryos. Data represent the mean ± SD of 4-7 independent experiments. Data were analyzed by ANOVA followed by Dunnet’s post-hoc testing, * p<0.05 versus untreated control cultures.

To investigate the selective contribution of mGluR5 to either necrotic and/or apoptotic cell death induced by OGD, we exposed cortical astrocytes derived from either wild-type (mGluR5+/+) or mGluR5 knock-out (mGluR5-/-) mice to OGD for 6-24 h. Apoptotic cell death induced by OGD treatment was reduced in astrocytes that lacked mGluR5, as assessed by Hoechst-stained condensed nuclei (Figure 4A). A two way ANOVA analysis demonstrated that the two genotypes were responding differently to OGD treatment for 6-24 h followed by a 24 h reperfusion treatment (Figure 4B). Specifically, the mGluR5 null genetic background provided significant protection against OGD-mediated apoptosis (Figure 4B). OGD treatment also resulted in increased necrotic cell death with time, as measured by increased LDH release (Figure 4C). However, necrotic astrocytic cell death in response to OGD was not attenuated in cortical mouse cultures derived from mGluR5-/- mouse embryos (Figure 4C). Taken together, these observations suggested a role for mGluR5 signaling in apoptotic, but not necrotic, astrocyte cell death following OGD.

### OGD induced apoptosis requires mGluR5-dependent InsP3 receptor activation

**Figure 5:**
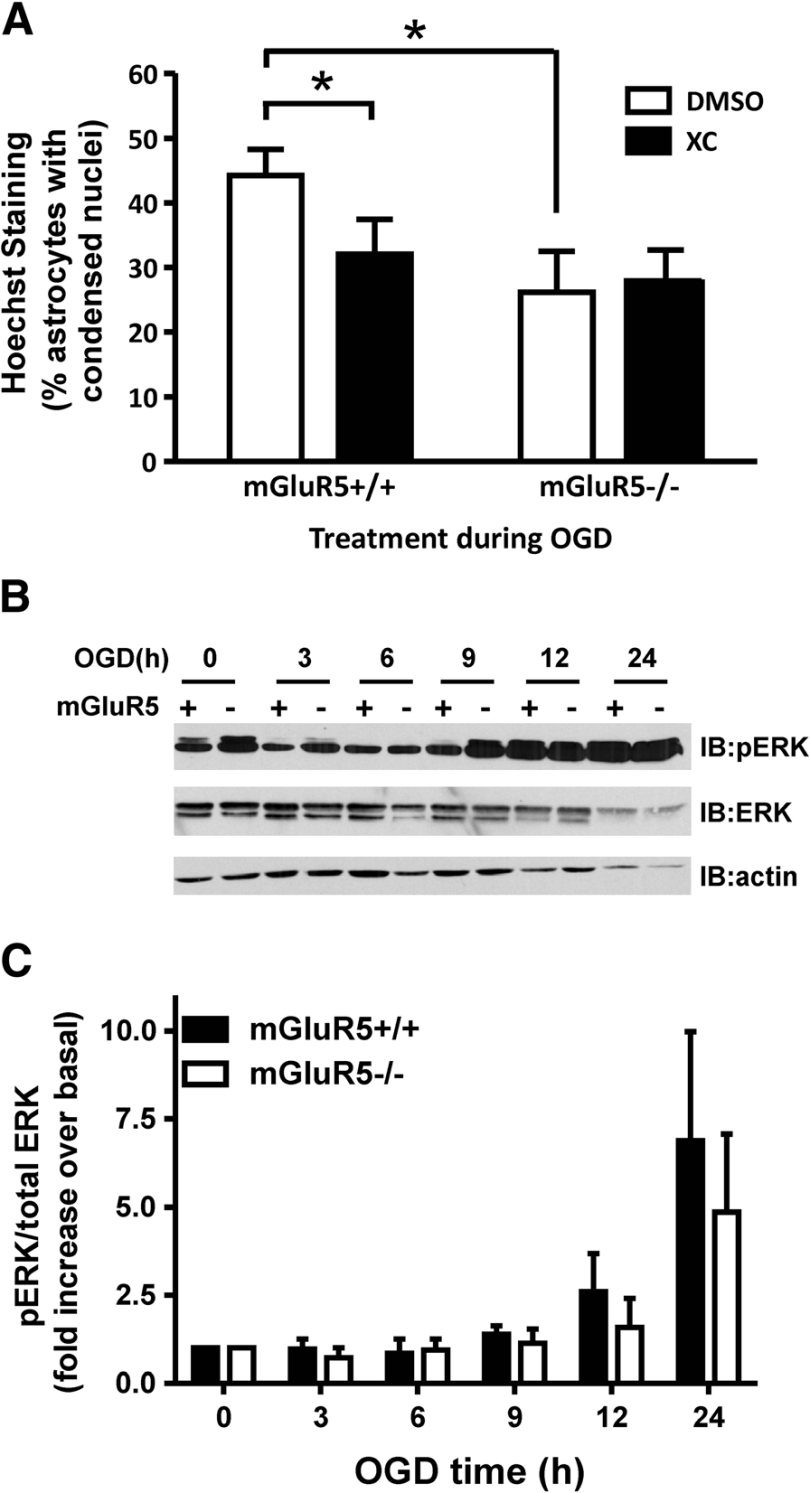
**Contribution of mGluR5 expression to OGD-induced InsP formation and ERK1/2 phosphorylation. A**) Quantification of apoptotic death of astrocytes derived from mGluR5-/- and mGluR5+/+ embryos in absence (DMSO) and presence 3 μM of the selective IP3 receptor inhibitor Xestospongin C (XC) in response to 12 h OGD followed by 24 h re-oxygenation. Bonferroni post-hoc test was performed on three independent experiments,* p<0.05 versus mGluR5+/+ DMSO treated control. **B**) Representative phosph-ERK1/2, total ERK1/2 and actin blots of primary mouse cortical astrocytes derived from mGluR5+/+ (+) and mGluR5-/- (-) embryos following 0-24 h OGD followed by 24 h re-oxygenation treated. **C**) Quantification of ERK1/2 phosphorylation in primary mouse cortical astrocytes derived from mGluR5+/+ (+) and mGluR5-/- (-) embryos following 0-24 h OGD followed by 24 h re-oxygenation treated. Data was normalized to total ERK1/2 protein expression. Data represents the mean ± SD of 4 independent experiments.

To further delineate whether the observed increase in InsP formation in response to OGD was dependent upon mGluR5 expression, we tested whether the antagonism of the IP3 receptor with the InsP3 receptor-specific inhibitor Xestospongin C would alter OGD induced apoptosis of astrocytes derived from wild-type and mGluR5-/- embryos. We found that Xestospongin C treatment significantly reduced the apoptotic cell death of wild-type astrocytes following 12 h of OGD treatment, but did not affect OGD-induced apoptosis of astrocytes derived from mGluR5-/- mice (Figure 5A). Furthermore, as shown previously (Figure 4B), the OGD-induced apoptosis of mGluR5-/- astrocytes was significantly reduced when compared to wild-type cultures and was not significantly different from wild-type cultures treated with Xestospongin C (Figure 5A). In contrast, the OGD time-dependent increase in ERK1/2 phosphorylation was not attenuated in astrocytic cultures derived from mGluR5-/- mice (Figure 5B and C). Taken together, these results further support the pharmacological data indicating that mGluR5 contributed to OGD-induced apoptosis as the consequence of InsP3 receptor activation, but does not contribute to increases in ERK1/2 phosphorylation.

### Role for Homer in OGD mediated astrocytic cell death

**Figure 6:**
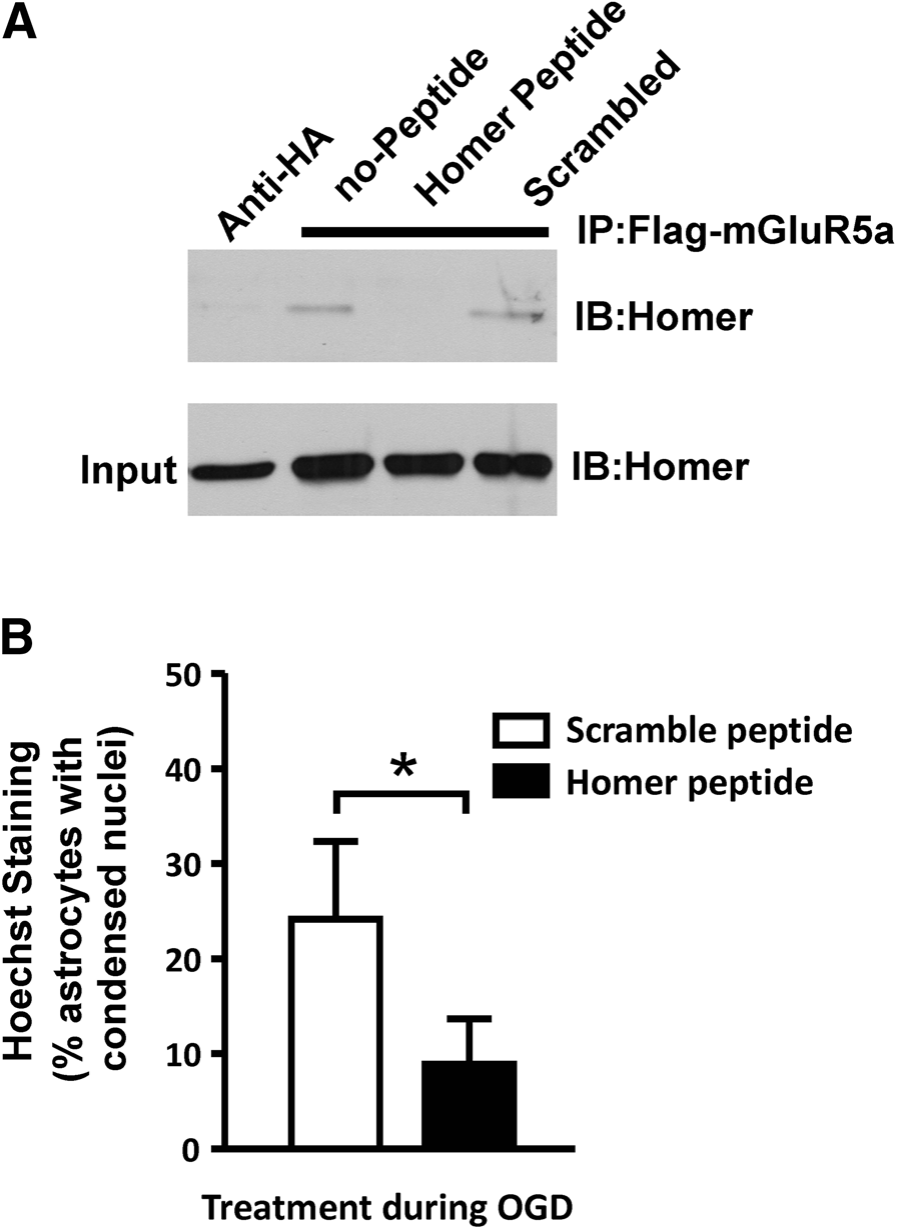
**Effect of disrupting mGluR5/Homer interactions on OGD-induced astrocytic cell death. A**) Representative immunoblot of Homer1b co-immunoprecipitation with Flag mGluR5a from HEK 293 cells transfected with plasmid cDNAs encoding Homer 1b (1 μg) and Flag-mGluR5a (3 μg) and treated with and without Homer (35 μM) and scrambled (35 μM) Tat-tagged peptides for 1 h. **B**) Quantification of apoptotic CD1 astrocytic cell death following 12 OGD and 24 h re-oxygenation following the treatment of the astrocytic cultures during the OGD period with Homer and scramble tat-tagged peptides (35 μM). Data represents the mean ± SD of 6 independent experiments. Asterisks represent significant differences by students t test, p<0.05.

It has been suggest that Homer proteins may regulate the efficiency of mGluR5-mediated activation of the InsP3 receptor [21], therefore we tested whether the treatment of astrocytes with a cell permeable Tat-tagged peptide corresponding to the mGluR5 Homer binding domain (Homer peptide) would attenuate OGD-induced astrocytic cell death [19]. Human embryonic kidney (HEK 293) cells were transfected with Flag-mGluR5a and Homer 1b to confirm that the Homer peptide disrupted mGluR5a/Homer 1b interactions by co-immunoprecipitation. We found that treatment of transfected HEK 293 cells with Homer peptide (35 μM), but not a scrambled peptide for 1 h antagonized the co-immunoprecipitation of Homer 1b with Flag-mGLuR5a (Figure 6A). Subsequently, the treatment of primary mouse cortical astrocytes with Homer peptide resulted in the reduction of astrocytic apoptotic cell death, when compared to astrocytes treated with scrambled peptide (Figure 6B). Thus, mGluR5a/Homer interactions appeared to also contribute to mGluR5-mediated apoptotic cell death following OGD treatment.

## Discussion

In the present study, we show that OGD induces both apoptotic and necrotic astrocytic cell death. We find that similar to what is observed for both acute and chronic neurodegenerative conditions, such as pain, amyotrophic lateral sclerosis, multiple sclerosis, and epilepsy [22-25,31-33] OGD also results in an increase in mGluR5a expression. We find InsP formation in cortical astrocytes is increased following OGD and that the treatment of cultures with the mGluR selective antagonist MPEP, not only attenuates OGD-mediated InsP formation, but the genetic deletion of mGluR5 selectively protects against OGD-induced apoptosis. The effects of mGluR5 are independent of the activation of the ERK1/2 signaling pathway, but appear to be associated with increased signaling via the PLC-InsP3-calcium pathway and Homer protein interactions.

We show that the mGluR5a expressed in response to injury are functional and activated after the ischemic insult. Group I mGluRs are known for their high constitutive and agonist-independent activity when they are overexpressed in heterologous systems [34]. However, in this case, because OGD-induced mGluR5 expression in astrocytes is not as high as those found in neuronal cultures or in transfected cells [20], increased mGluR5 activity may also be due to glutamate released by astrocytes in response to ischemia. Indeed, ischemic conditions could result in glutamate release by astrocytes through a variety of mechanisms [35]. The observed increases in InsP formation following OGD are consistent with previous work demonstrating that OGD results in increased InsP formation, likely via a metabotropic glutamate receptor [36].

We observe that OGD treatment activates a time-dependent increase in ERK1/2 phosphorylation that appears to be independent of mGluR5 activity, as increases in ERK1/2 phosphorylation were not antagonized by treatment of cultures with the mGluR5 selective antagonist MPEP. Furthermore, the genetic deletion of the mGluR5 gene did not result in diminished ERK1/2 phosphorylation in response to OGD. Indeed, in contrast to the InsP3 pathway, the mGluR5-induced activation of MAPK pathway occurs via mechanisms that are largely independent of PLC activation and calcium release in both striatal neurons (Homer protein dependent) and astrocytes (EGF receptor transactivation) [19,37,38]. The OGD-induced increase in ERK activation in astrocytes, as previously reported [39], did not significantly change in presence of the mGluR5 antagonist MPEP. Thus, it is possible that glutamate released in response to OGD may activate alternative mGluR subtypes that may be directly linked to the activation pro-survival signal transduction pathways in astrocytes. Consistent with this, mGluR3 is implicated in protecting against astrocytes apoptosis following OGD [40].

Interestingly, the mGluR5-dependent activation of PLC-mediated signaling leads to excessive calcium release and excitotoxic striatal neuronal death, which could be prevented by the down regulation of Group I mGluR expression following estradiol treatment [18]. Thus, a selective increase in PLC signaling likely correlates with a pro-apoptotic role for astrocytic mGluR5, as OGD-induced apoptosis is reduced in astrocytes derived from mGluR5 null embryos. Moreover, selective mGluR5 blockade has been reported to be neuroprotective [41]. Thus, it appears that increased mGluR5 signaling in astrocytes might lead to cell death following OGD-increased mGluR5a protein expression levels. Interestingly, mGluR5 has been shown to be upregulated in the spinal cord of ALS patients [22,24] and astrocytic cell death in an amyotrophic lateral sclerosis transgenic model has been shown to be attenuated by blocking the mGluR5 receptor *in vivo*[42]. Moreover, the disease onset was also delayed by this treatment. Similarly, the brain permeable mGluR5 antagonist MTEP provides neuroprotection in an *in vivo* ischemic model [17].

Because the effect of the InsP3 receptor inhibitor Xestospongin C on apoptosis is not observed in astrocytes lacking the mGluR5, we have confirmed that the mGluR5-PLC-InsP3-calcium signaling pathway is responsible for induction of apoptosis by releasing calcium from intracellular stores. As reviewed by Hanson et al. [43], calcium released through InsP3 receptors is sequestered by mitochondria and mitochondrial calcium overload can induce the apoptotic cascade. In addition, calcium release can also stimulate calcium-sensitive enzymes such as the phosphatase calcineurin, which is involved in other apoptotic mechanisms [43]. Furthermore, we show that the formation of a molecular complex between Homer proteins and mGluR5 contributes to the activation of a pro-apoptotic pathway, as the treatment of astrocytes with a Tat-Homer interference peptide reduced OGD-induced astrocytes apoptosis. This Tat-peptide was previously shown to block mGluR5/Homer interactions but also blocks Homer-dependent activation of ERK1/2 [19]. Thus, although we did not observe reduced ERK1/2 phosphorylation in response to OGD following either MPEP treatment or the loss of mGluR5 expression, it is possible that mGluR5-dependent activation of the ERK1/2 pathway via a Homer-dependent mechanism may also function to antagonize mGluR5-mediated apoptosis of astrocytes that is induced as the consequence of PLC-mediated mGluR5 signaling.

In conclusion, because of the neuroprotective role of astrocytes, astrocytic cell death could have a huge impact on acute or chronic neurodegenerative conditions. Thus, targeting mGluR5-signaling pathway involved in the induction of astrocyte apoptosis may have therapeutic benefits following ischemic insults such as stroke. For example, selective blockade of mGluR5-dependent PLC signaling in astrocytes might help limit cellular loss in a variety of neurodegenerative diseases. In contrast, the selective activation of mGluR5 G protein-mediated PLC signaling might promote apoptotic cell death in brain tumors, such as astrocytomas. This is of particular importance considering that mutations in mGluR1a have been associated with a variety of tumors, and that many of the mutations bias receptor signaling via the activation of ERK1/2 at the expense of G protein signaling [44-46] suggesting it may be possible to design biased allosteric agonists to prevent both astrocytic and neuronal cell death.

## Methods

### Materials

2-Methyl-6-(phenylethynyl) pyridine hydrochloride (MPEP) was purchased from Tocris Cookson (Ellisville, MO). All cell culture reagents were from Invitrogen (Burlington, ON). Cytotoxicity detection kit plus (lactose dehydrogenase, LDH) was from Roche (Mississauga, ON). Rabbit anti-mGluR5 antibodies were from Millipore (Billerica, MA). Rabbit anti-phospho-p44/42ERK and anti-p44/42 ERK antibodies were from Cell Signaling Technology (Danvers, MA). Rabbit anti-actin antibody was from Santa Cruz Biotechnology (Santa Cruz, CA). Horseradish peroxidase-conjugated anti-rabbit antibody, Dowex 1-X8 (formate form) resin with 200–400 mesh and Bradford assay were purchased from Bio-Rad (Mississauga, ON). Anti-mouse IgG secondary antibody and ECL Western blotting detection reagents were from GE Healthcare (Oakville, ON). *myo*-^3^H]Inositol was acquired from PerkinElmer Life Sciences (Waltham, MA). The QuantiTect SYBR green single-step RT-PCR was from Qiagen (Toronto, ON). Alexa-488 anti-mouse was purchased from Molecular Probes (Burlington, ON). GenElute Mammalian Total RNA miniprep kit, Hoechst 33342, (3-(4,5-Dimethylthiazol-2-yl)-2,5-diphenyltetrazolium bromide (MTT), Xestospongin C, and all other biochemical reagents were purchased from Sigma-Aldrich (St. Louis, MO). The Tat-peptides corresponding to the mGluR5 binding motif for Homer proteins (Homer peptide: YGRKKRRQRRRALTPPSPFRDS and Scramble peptide: YGRKKRRQRRRTRSLPSDPPAF [19] were purchased from CanPeptide (Pointe Claire, PQ).

### Animals

CD1 and BL6/129-Grm5tm1Rod/J [47] mice were purchased from Charles River Labs and Jackson Laboratory, respectively. Mice were housed in an animal care facility at 23°C on a 12 h light/12 h dark cycle with food and water provided *ad libitum*. Animal care was in accordance with the University of Western Ontario Animal Care Committee.

### Primary astrocyte cultures

Cortices were dissected from CD1 or BL6/129Grm5 individual littermate embryos (E15-16) and separated from the striatum, as previously described [20]. After dissociation in D-MEM/glucose/10%FBS/Gentamycin/L-glutamine media, the cells were plated onto poly-D-lysine coated culture flasks and maintained at 37°C and 5%CO_2_. Plating media was replaced by fresh media the following day and every 4 days thereafter. At DIV 7, the primary cultures were shaken overnight at 260 rpm at 37°C and washed three times with PBS to remove microglia. Cells were allowed to recover in fresh media and after 24 hours cells were trypsinized and re-plated onto poly-D-lysine coated plates at a density of 12,000 cells/cm^2^. Experiments were performed at DIV 14-19. The purity of the astrocyte cultures was assessed by immunocytochemistry with mouse anti-GFAP (1:400) and Alexa-488 anti-mouse (1:500) by microscopy (with nuclear staining with Hoechst 33258) as adapted from [48].

### Oxygen/glucose deprivation experiments

After one wash with glucose/ L-glutamine-free D-MEM, astrocyte cultures were incubated for different periods of time with the same media in a hypoxic chamber (COY Laboratory Products Inc.) at 37°C, 5% CO_2_ and 0% O_2_, as described previously [40]. Control cell plates (0 h) were maintained in normoxic conditions. After OGD, equal volume of DMEM/2X glucose/10%FBS/Gentamycin/L-glutamine media was added to the cells. The media of control astrocyte cultures was replaced by equal volumes of D-MEM with 2X glucose and without glucose. The cells were incubated at 37°C, 5% CO_2_ for 24 h (reperfusion).

### Western blotting

Cells were harvested either immediately after the incubation in the hypoxic chamber (OGD), or after the 24 h reperfusion period with RIPA buffer (10 mM Tris-HCl pH=7.5, 140 mM NaCl, 1% Nonidet P-40, 1% sodium deoxycholate, 0.1% SDS). After solublization, insoluble material was removed by centrifugation and supernatant was used to determine protein concentration by Bradford assay. After dilution in SDS sample buffer, 75-100 μg of protein was loaded into a 10% acrylamide SDS-PAGE gel. After electrophoresis, proteins were transferred to a nitrocellulose membrane. After blocking non-specific binding with 10% non-fat milk in TBS with 0.05% Tween-20 (TBS-T), membranes were incubated with rabbit anti-mGluR5 (1:4000), rabbit anti-actin (1:10000), rabbit anti-phospho-pERK44/42 (1:1000), or rabbit anti-pERK44/42 (1:1000, after anti-phosphoERK stripping) antibodies overnight at 4°C. After three washes with TBS-T, blots were incubated 1 hour at room temperature with HRP-conjugated anti-rabbit (1:10000) or anti-mouse (1:5000) antibodies [20]. After three washes with TBS-T, blots were developed with ECL and exposed to films.

### Quantitative real time reverse-transcript polymerase chain reaction (qRT-PCR)

RNA samples were isolated with the GenElute Mammalian Total RNA miniprep kit and treated with DNAse I following the manufacturer instructions. 30 ng of total RNA was used per reaction and mouse mGluR5 mRNA and the mitochondrial ribosomal protein S12 (endogenous housekeeping gene) mRNAs were amplified separately using the following primers: 5^′^ mGluR5: 5^′^GGTGGTAGCCTGCTTCTGTGA3^′^ (exon2), 3^′^mGluR5: 5^′^CGCTGATACCCATCTGTCACG3^′^ (exon3), 5^′^S12:, 5^′^GGAAGGCATAGCTGCTGG3^′^, 3^′^S12: 5^′^CCTCGATGACATCCTTGG3^′^ using the QuantiTect SYBR green single-step RT-PCR kit as per manufacturers’ instructions. The comparative Ct method (2–[delta][delta]Ct) was used for quantification [49].

### Inositol phosphate formation assay

Inositol lipids were radiolabeled by incubating the cortical astrocyte cultures overnight with 1 μCi/ml *myo*-^3^H]inositol in D-MEM, as previously described by Ribeiro et al. [20]. In brief, incorporated *myo*-^3^H]inositol was removed by washing cells with Hank’s balanced salt solution (HBSS). Cells were transferred in D-MEM without glucose and placed in the hypoxic chamber or not (0 h OGD control) for 4 h. After ischemia, LiCl was added to the wells to achieve a final concentration of 10 mM and the cells were incubated for 30 min in presence or in absence of 10 μM MPEP. The reactions were stopped on ice by the addition of 500 μl of perchloric acid and then neutralized with 400 μl of 0.72 M KOH, 0.6 M KHCO3. Total ^3^H]inositol incorporated into cells was determined by counting the radioactivity present in 50 μl of cell lysate. Total inositol phosphate was purified from cell extracts by anion exchange chromatography using Dowex 1-X8 (formate form) 200–400 mesh anion exchange resin. ^3^H]Inositol phosphate formation was determined by liquid scintillation.

### MTT cell viability assay

After reperfusion, 1/10 of media volume of 5 mg/ml MTT was added to the culture dishes and incubated at 37°C, 5% CO_2_ for 3 h. The reaction was then stopped by adding an equal volume of isopropanol. After trituration, the crystal deposits (converted dye) were dissolved by shaking for 20 min. Absorbances at 570 nm and 690 nm (background) were read in triplicate and absorbance values were corrected for the absorbance due to the culture media in absence of cells [50].

### Hoechst staining

As described by Steckley et al., [49] Hoechst 33342 dye was added in triplicate directly to live cells and incubated for 30 min at 37°C. The media was then removed and the cells fixed in 3.7% formaldehyde for 60 min at RT. After three PBS washes, the cells were kept at 4°C until imaged and apoptotic cells (condensed nuclei) were quantified using ImageJ software. For each experimental data point, 1000-2000 cells were counted from triplicate wells of sister cultures from at least 3 independent experiments.

### LDH release assay

After OGD/reperfusion experiments, lysis buffer (10 mM Tris-HCl pH=7.5, 140 mM NaCl, 1% Nonidet P-40, 1% sodium deoxycholate, 0.1% SDS) was added to culture wells to determine the maximum LDH release. After 15 min incubation at 37°C and 5% CO_2_, 50 μl aliquots of triplicate samples or cell-free culture media (background) were transferred to wells of a 96 well plate containing 50 μl of PBS/well and experiments were performed according to the manufacturer instructions (Cytotoxicity detection kit plus, Roche). LDH release was determined by absorbance (A_492 nm_-A_690 nm_) measured using a SPECTRAmax M5 plate reader and expressed as percentage of the maximum release.

### Data analysis

GraphPad Prism software was used to analyze data for statistical significance and for curve fitting. Statistical significance was determined by analysis of variance (ANOVA) testing followed by post-hoc Multiple Comparison testing.

## Abbreviations

ERK: Extracellular regulated kinase; LDH: Lactose dehydrogenase; InsP: Inositol phosphate; mGluR: Metabotropic glutamate receptor; MPEP: 2-Methyl-6-(phenylethynyl) pyridine hydrochloride; MTT: (3-(4,5-Dimethylthiazol-2-yl)-2,5-diphenyltetrazolium bromide; OGD: Oxygen glucose deprivation; PLC: Phospholipase C.

## Competing interests

The authors declare that they have no competing interests.

## Authors’ contributions

MP, FMR, JG, and JLE performed the experiments included in the manuscript. LBD and TC provided technical assistance with cell cultures and confocal microscopy. SPC and SSGF conceived of the experiments and wrote the manuscript. All of the authors have read the manuscript.
